# The effect of transcranial direct current stimulation on the expression of the flexor synergy in the paretic arm in chronic stroke is dependent on shoulder abduction loading

**DOI:** 10.3389/fnhum.2015.00262

**Published:** 2015-05-11

**Authors:** Jun Yao, Justin Drogos, Fleur Veltink, Caitlyn Anderson, Janny Concha Urday Zaa, Laura Imming Hanson, Julius P. A. Dewald

**Affiliations:** ^1^Department of Physical Therapy and Human Movement Sciences, Northwestern UniversityChicago, IL, USA; ^2^The Medical Faculty of the Radboud UniversityNijmegen, Netherlands; ^3^Department of Biomedical Engineering, Northwestern UniversityChicago, IL, USA; ^4^Department of Physical Medicine and Rehabilitation, Northwestern UniversityChicago, IL, USA

**Keywords:** transcranial direct current stimulation, reaching arm movements, stroke, flexion synergy, motor control disorders

## Abstract

Reaching ability of the paretic upper extremity in individuals with stroke decreases with increased shoulder abduction (SABD) loads. Transcranial direct current stimulation (tDCS) has been implemented to improve movement ability following stroke. However, results from previous studies vary, perhaps due to the influence of impairment level and the type of motor tasks that were used to study the effects of tDCS. This study specifically examines the impact of SABD loading on the effects of tDCS in 9 individuals with moderate to severe chronic stroke. In 3 different sessions, participants repeated a reaching assessment with various SABD loads (supported on a haptic table, 25%, and 50% of maximum voluntary SABD torque) in random order, pre and post one of the following 15-min tDCS protocols: anodal stimulation of lesioned M1, cathodal stimulation of non-lesioned M1, or anodal stimulation of non-lesioned M1. Sham stimulation was also conducted preceding one of the tDCS sessions. The averaged maximum reaching distance over valid trials was calculated for each condition. We observed significant interactions between SABD load, tDCS protocol and time (i.e., pre or post-tDCS). *Post hoc* test showed that anodal stimulation of the lesioned M1 caused a clear trend (*p* = 0.058) of increasing the reaching ability at a medium level of SABD loading (25%), but not for higher loads (50%). This suggests that anodal stimulation increases residual corticospinal tract activity, which successfully increases reaching ability at moderate loads; however, is insufficient to make significant changes at higher SABD loads. We also found that cathodal stimulation of the non-lesioned M1 significantly (*p* = 0.018) decreased the reaching distance at a high level of SABD loading (50%). This study demonstrated, for the first time, that the effect of tDCS on the reaching ability is dependent on SABD loads in individuals with moderate to severe stroke.

## Introduction

Stroke is the leading cause of disability in the United States (Roger et al., 2012). Despite novel interventions developed in recent years that emphasize task-specific repetition and increased intensity, only 20% of individuals regain normal arm function 3 months post-stroke (Kwakkel et al., 2003). In particular, in individuals with moderate to severe arm impairment, the flexion synergy (i.e., the obligatory coupling between shoulder abduction (SABD) and elbow flexion) causes decreased reaching ability of the paretic upper extremity when increased SABD loading is applied to the paretic arm (Sukal et al., 2007). Therefore, many stroke survivors report devastating limitations with activities of daily living often related to paretic limb dysfunction.

In order to increase motor function, transcranial direct current stimulation (tDCS) has been tested in individuals with different pathological conditions, including stroke. However, previous results of tDCS in individuals with stroke vary. Some studies showed significantly higher gains in outcome measures in the groups who underwent real tDCS as compared to sham tDCS (Kim et al., 2010; Lindenberg et al., 2010; Bolognini et al., 2011), while other studies found no between-group differences (Hesse et al., 2011; Rossi et al., 2013). Furthermore, one of the previous studies showed that effects of tDCS were different in mildly vs. severely impaired individuals (Bradnam et al., 2012). These results suggest that the effects of tDCS may be affected by between-subject differences related to the amount of neural resources that are still available after a stroke. Consequently, although never studied, within-subject differences related to the amount of neural resources that are required for different motor tasks may also change the effects of tDCS in individuals with stroke.

More specifically, in individuals with moderate to severe motor impairments following a stroke, preliminary evidence reported an increased reliance on projections from the non-lesioned hemisphere (Dewald et al., 1995; Ellis et al., 2009). In humans, projections from the non-lesioned side to the ipsilateral paretic upper limb motor neurons mostly occur via cortico-reticulospinal tracts (CRSTs; Schwerin et al., 2008, 2011). In non-human primates, the reticulospinal tracts (RSTs) branch widely at different segments of the spinal cord, facilitating ipsilateral flexion and suppress extension (Davidson and Buford, 2004, 2006; Baker, 2011). This is presumably also the case in humans. Due to these properties of RSTs, muscle recruitment via RSTs is expected to result in obligatory coupling of shoulder abductor and elbow/wrist and fingers flexors, i.e., causing the flexion synergy in an individual with stroke.

In order to increase motor performance, anodal tDCS (a-tDCS) over the lesioned hemisphere has been previously studied (Cuypers et al., 2013). A-tDCS over the lesioned hemisphere is expected to facilitate corticospinal activity in the remaining tracts (Cuypers et al., 2013). As a result, a decreased usage of CRSTs from the non-lesioned side is anticipated to reduce the expression of the flexion synergy. However, when more intense motor tasks require for the use of much more neural resources than all remaining CST from the lesioned hemisphere, CRSTs from the non-lesioned side become the primary neural resource for muscle recruitment. In this circumstance, the facilitatory effects of a-tDCS may be negligible and no longer apparent. Therefore, we hypothesize that a-tDCS will successfully increase reaching distance while generating moderate levels of SABD loading, but not at higher levels.

Cathodal tDCS (c-tDCS) of the non-lesioned hemisphere is hypothesized to reduce interhemispheric inhibition on the lesioned side, thus releasing the residual CSTs from the lesioned side (Krause et al., 2013). This seems to be true in mildly impaired individuals; however, for more severely impaired individuals, this may no longer hold (Bradnam et al., 2012). This effect of c-tDCS as shown in mildly impaired individuals is in agreement with the ill-defined role of the non-lesioned hemisphere. As discussed previously, increased reliance on the non-lesioned hemisphere may cause abnormal movement patterns, specifically the flexion synergy (Chen et al., 2014), which is a maladaptation that should be avoided. However, increased reliance on the non-lesioned hemisphere can also be an important compensation against total paralysis in the paretic upper limb following stroke, especially in more severely impaired individuals who are in short supply of cortical neural input from the lesioned hemisphere. When these individuals perform motor tasks that require greater SABD loads, contribution from the non-lesioned cortex is likely to be at its maximum. In this case, the effect of cortical stimulation-induced facilitation of residual CSTs may be too small to be noticeable. Therefore, we hypothesize that c-tDCS of the non-lesioned hemisphere will fail to increase the reaching distance with a high SABD loading in individuals with moderate to severe stroke.

Effects of a-tDCS applied to the non-lesioned hemisphere have not been widely studied before. The possible effects of a-tDCS over non-lesioned hemisphere include increased cortical activity from the non-lesioned side, and possibly an increased inhibition from the non-lesioned side to the lesioned side. However, considering that recruitment of neural resources from the non-lesioned side is already close to its full capability in individuals with moderate to severe stroke, we hypothesize that a-tDCS of the non-lesioned hemisphere has no significant effect on the expression of the flexion synergy.

In short, this study aims to test the impact of SABD loading on the effects of tDCS on reaching distance in individuals with moderate to severe chronic stroke.

## Methods

### Subjects

Ten individuals with chronic stroke participated in this research. One individual was excluded in the analysis, because he was unable to get into the initial arm position required for our protocol. Furthermore, subject number 9 only finished 2 sessions (a-tDCS of lesioned side and c-tDCS of the non-lesioned side) and missed the last session (i.e., the a-tDCS of non-lesioned side) due to surgery. Subject information is listed in Table 1. All individuals were screened for inclusion by a licensed physical therapist. Exclusion criteria include a Fugl Meyer Assessment (FMA) score of the upper limb above 40 or below 10 out of 66 (Fugl-Meyer et al., 1975; Gladstone et al., 2002), history of seizures, acute eczema, shoulder pain, visual deficits, pregnancy, metallic implants near the tDCS electrodes, cardiac pacemaker or other implanted devices, known adverse reactions to applications of electrical current, or other unstable medical conditions. This study was ethically approved by the Northwestern University institutional review board, and all subjects participated voluntarily and gave informed consent.

**Table 1:** **Characteristics of the analyzed subjects**.

Subject	Age (years)	Sex	FMA score*	Paretic side	Month and year stroke
1.	62	M	40	Right	Feb 2007
2.	70	M	24	Left	July 2001
3.	61	F	20	Right	Aug 2007
4.	57	M	24	Left	Dec 2006
5.	43	M	28	Left	March 2010
6.	78	M	22	Left	March 2008
7.	66	M	39	Left	Dec 2002
8.	55	M	22	Right	Aug 2010
9.	48	M	39	Left	June 1998
*N* = 9	60 ± 10.8	M/F: 8/1	28.7 ± 8.3	L/R: 6/3

M, Male; F, female; L, left; R, right; *Upper Extremity Fugl-Myer Motor Assessment, scale 0–66. Bottom row, group means ± 1 SD.

### Experimental Setup

The experimental setup consisted of an isometric setup, to measure subject-specific maximal voluntary SABD torques, a haptic master robot to modulate the shoulder loads applied to the paretic arm, and a tDCS stimulator.

#### Isometric Measurement Setup

A Biodex chair (Biodex Inc, Ser no 220297650, Shirley, NY) with a 6-degree of freedom JR3 load cell was used to measure the maximum SABD torque under isometric conditions. In the isometric setup, each individual sat on the Biodex chair with straps across the shoulder and waist to immobilize the trunk. Individual’s forearms were casted from the proximal forearm to the fingers, and then firmly attached to a JR3 load cell. The arm was placed in a position of 90° SABD, 40° shoulder flexion, and 70° elbow flexion.

#### ACT3D Robot Setup

**Figure 1:**
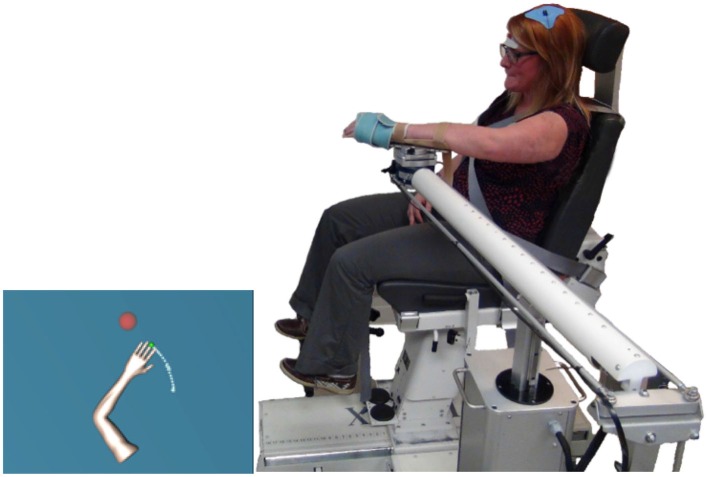
**ACT3D robot setup.** On the left, it shows the visual feedback provided to the participant. On the right, it shows the ACT3D robot setup and the c-tDCS setup.

All the participants performed the reaching task in the Arm Coordination Training 3D device (ACT3D robot, Moog-FCS Bv, Netherlands) (Figure 1). In addition to measuring the location of the hand, the ACT3D robot allows for increasing or decreasing SABD loads during reaching tasks by imposing a vertical force on the forearm (Sukal et al., 2007).

During data collection, each individual sat on the Biodex chair with trunk secured. The forearm rested and was attached to an orthosis connected to the robot. The initial arm position (the home position) was set to 90° SABD, 40° shoulder flexion and 70° elbow flexion to match the position used during isometric testing. The lengths of the upper arm, lower arm and hand were measured to provide an avatar of the upper limb on a flat screen placed in front of the subject. The initial visual feedback included an avatar of the arm and a ball for the home position. After staying in the home position for 5 s, a new ball appeared to cue the subject to reach toward the target position (resulting in a 90° SABD, 110° shoulder flexion and 0° elbow flexion if the target was reached by the participant). For SABD trials, the participant had to lift their arm off of the table (which was lowered to 80° SABD) into the home position (90° SABD) and maintain their arm off of the table while reaching for the target.

#### tDCS Setup

Transcranial direct current stimulation was delivered using a current controlled dual channel iontophoresis system (Dupel, Hannover Germany and Empi, Clear Lake, USA) via two distilled water-soaked electrodes with an active area of 16 cm^2^ (Dupel, type Large Blue) with a dosage of 0.8 mA lasting 15 min. The current density was therefore 0.05 mA/cm^2^, which was in the similar range as many other t-DCS studies, as reviewed by Bastani and Jaberzadeh (Bastani and Jaberzadeh, 2012). The active electrode was positioned over the non-lesioned M1 or lesioned M1 areas, depending on the protocol being tested. All stimulation conditions had a reference electrode on the supraorbital region of the forehead. Sham stimulation was applied using one of the previously mentioned configurations, and the current intensity was ramped down to zero after 30 s (Schlaug and Renga, 2008; Bradnam et al., 2012).

### Experimental Protocol

In each individual, three experimental sessions were performed in random order on three different days, at least 2 weeks apart. During the first session the maximum SABD force was measured using the isometric setup. Then subject was moved to the ACT3D robot setup to perform the required reaching task from the home position to the target position, 10 trials for each of the three SABD loads (haptic surface (table), 25% and 50% of maximal voluntary torque (MVT) of SABD, heretofore referred to as MVT_25 and MVT_50). Reaching trials were separated into two sets of five reaches for each loading condition. One set of reaches of the table condition was conducted first to establish a baseline, and then the remaining sets were performed in random order. Participants were instructed to reach slowly to the target to minimize possible impact of spasticity and maximize the reaching distance (Kamper et al., 2002). Participants were given at least a 20-s resting period between trials, and a 1-min resting period between sets in order to minimize fatigue. After finishing pre-tDCS reaching test, the participants received either a-tDCS on the lesioned side, c-tDCS on the non-lesioned side or a-tDCS on the non-lesioned side, with the order randomly selected for each of the participants. Immediately after stimulation, a post-stimulation set of reaching tasks was completed within 30 min. In one of the three real stimulation sessions, sham stimulation and a post sham-evaluation was included before the real stimulation was provided.

### Data Analysis

Collected kinematic data were processed in Matlab (The Mathworks; Natick, Massachusetts). Maximal reaching distance was calculated for each of the valid trials (invalid trials include trials where the tested arm touched the haptic table during loading of MVT_25 or MVT_50 conditions and trials that went outside the valid zone, i.e., ±45° with regards to the line from home position to the target position). The averaged maximal value of all the valid trials was then normalized by the theoretical maximum reaching distance that a participant should be able to generate given the measured arm length without considering the impact of stroke.

The normality of the data was confirmed using the Shaprio-Wilk test. Data skewness was further checked by the *Z*-score. If a *Z*-score was out of the range of ±1.96, the corresponding paired-data were corrected. Subsequently, a three-way repeated measures ANOVA was applied to test for significant effects, factors being time (pre- and post-tDCS), SABD conditions (table, MVT_25 and MVT_50 conditions) and the tDCS configurations (a-tDCS from lesioned side, c-tDCS from non-lesioned side, a-tDCS from non-lesioned side and sham conditions). A *post hoc* paired *t*-test was then used to find the significant effect of each significant factor (*p* < 0.05). A clear trend of significance (*p* < 0.1) was also reported.

## Results

**Figure 2:**
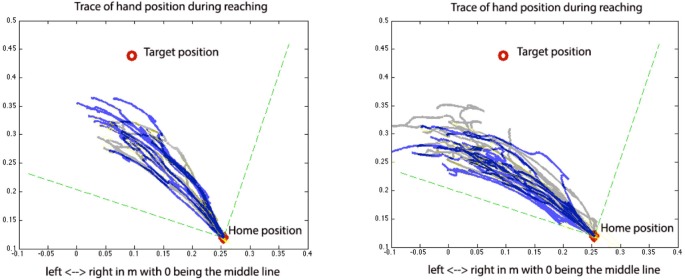
**Pre (gray) and post (blue) tDCS reaching trials for a-tDCS over the lesioned side with 25% of the MVT shoulder load (left) and c-tDCS over the non-lesioned side with 50% of the MVT shoulder load (right)**. In this figure, the gray and blue traces represent the traces of hand position pre- and post-tDCS, respectively.

An example of a single performance of reaching trials before and after a-tDCS over the lesioned hemisphere at MVT_25 condition and c-tDCS from the non-lesioned hemisphere at MVT_50 condition was plotted in Figure 2.

All the data were normally distributed, as determined by Shapiro-Wilk Test (the *p*-values were in the range of 0.082–0.958, all being non-significant). The *Z*-scores of all data were in the range of ±1.96 (absolute values are in the range of 0.005–1.402), except the normalized reaching distance for MVT_50 before the c-tDCS over the non-lesioned side (*Z*-score = −2.033). Based on these results, the normalized reaching distances without data correction were used in the repeated measures ANOVA to test possible significant effects of SABD loading (table, MVT_25, and MVT_50), tDCS configuration (a-tDCS over lesioned side, c-tDCS and a-tDCS over non-lesioned side, sham stimulation) and/or time (before and after stimulation). In correspondence with earlier studies (Sukal et al., 2007; Ellis et al., 2009), the 3-way repeated measures ANOVA reported a significant effect for SABD loading factor (*p* = 0.002, *F* = 10.785). Significant interactions between tDCS configuration and SABD loading (*p* = 0.019, *F* = 2.962), as well as among tDCS configuration, SABD loading and time (*p* = 0.019, *F* = 2.940) were also found, suggesting that the changes induced by tDCS on reaching distance were dependent on the SABD loading level and on the configuration of tDCS. No other significant effects or trends were reported by the repeated measures ANOVA.

**Figure 3:**
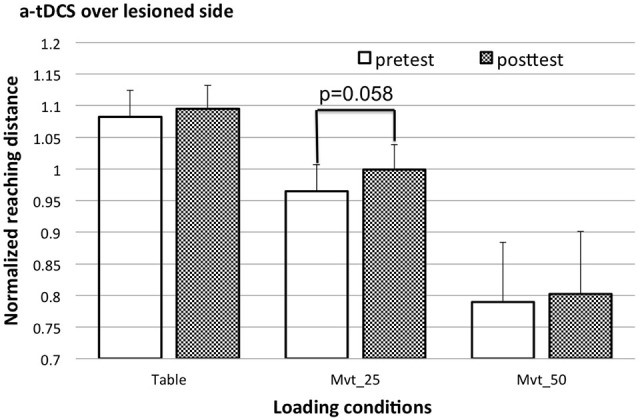
**The cross-subject mean and standard error of reaching distance pre (open bars) and post a-tDCS (shaded bars)**.

**Figure 4:**
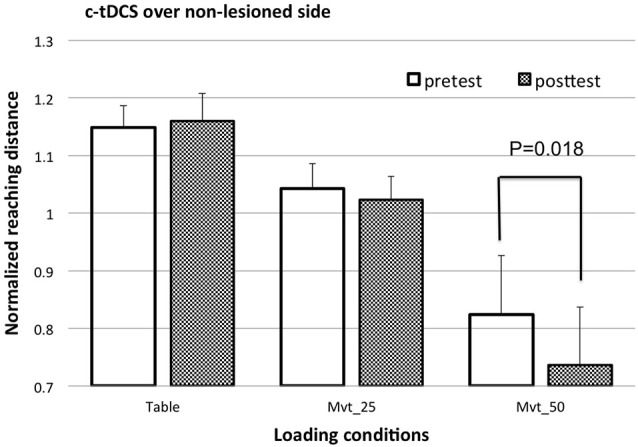
**The cross-subject mean and standard error of reaching distance pre (open bars) and post c-tDCS (shaded bars) over non-lesioned M1 area**.

Since significant interactions among tDCS configuration, SABD loading and time were found, the *post hoc* test using a paired *t*-test comparing the means of the reaching distance pre and post tDCS was conducted separately for each loading condition and each tDCS condition. Before *post hoc* testing, the normalized reaching distances for MVT_50 before and after c-tDCS of the non-lesioned side were corrected for data skewness. After correction, the *Z*-scores were −0.594 and −0.431, respectively. The paired *t-test* reported that a-tDCS over the lesioned M1 had a clear-trend in increasing the reaching ability at a moderate load (MVT_25) (*p* = 0.058), but not for the table or heavy load (MVT_50) conditions (see Figure 3 for the cross-participant means and standard errors of the reaching distance pre and post a-tDCS). The *post hoc* test also reported that c-tDCS over the non-lesioned M1 significantly decreased reaching ability at a high load (MVT_50) (after data correction *p* = 0.018, before data correction *p* = 0.04), but not at the table or moderate shoulder load (MVT_25) conditions (see Figure 4 for the cross-participant means and standard errors of reaching distance pre and post c-tDCS). No other significant results or clear-trends were found by *post hoc* testing.

**Figure 5:**
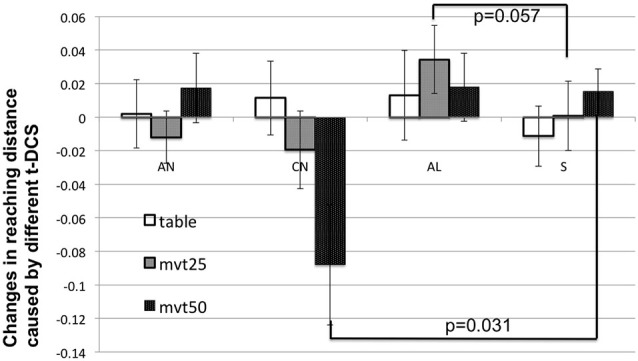
**The cross-subject mean and standard deviation of normalized tDCS-induced changes in reaching distance (i.e., (post-tDCS reaching distance − pre-tDCS reaching distance)/(pre-tDCS reaching distance)) with different SABD loadings**. In this figure, AN, CN, AL, and S mean a-tDCS of non-lesioned side, c-tDCS of non-lesioned side, a-tDCS of lesioned side and sham stimulation, respectively.

Additionally, a separate paired *t*-test was conducted to compare changes in reaching distance induced by the different real tDCS conditions vs. the sham condition, for each load level. As shown in Figure 5, this *post hoc* test reported that (1) changes caused by a-tDCS of the lesioned M1 showed a clear trend of increase of reaching distance with respect to the sham stimulation (*p* = 0.057), given a medium level of SABD loading (MVT_25); and (2) changes caused by c-tDCS of the non-lesioned M1 were significantly (*p* = 0.031) larger than those caused by the sham stimulation given a high level of SABD loading (MVT_50). No other significant results or clear trends were found by the *post hoc* test.

## Discussion

### Effects of a-tDCS of Lesioned Side on Reaching Distance with Various SABD Loadings

In general, in the recruited 9 individuals with moderate to severe stroke, a-tDCS of the lesioned side showed a clear trend (*p* = 0.058) of increasing the reaching distance when a moderate load (MVT_25) was applied to the impaired shoulder. As mentioned before, the *Z*-scores of the data sets before and after a-tDCS at the MCT_25 condition were in the range of ±1.96. Therefore, there is no strong reason to perform a data correction. However, by visually checking the histogram, we observed slightly longer positive tail. If we corrected these two data sets using Ln transform, then the *p*-value of the paired *t*-test dropped to 0.05. Furthermore, we closely looked at effects in conjunction with the degree of initial impairment for each subject. Specifically for the results pre and post a-tDCS of the lesioned hemisphere, all the subjects had an increased reaching distance post a-tDCS of the lesioned side, expect one subject who has the lowest FMA score (FMA = 20) among all the nine subjects. If this most severely impaired subject was excluded from the analysis, the paired *t-test* resulted in a *p* = 0.003. This result suggests that the effect of t-DCS may also depend on impairment level. Overall, the increased reaching ability after a-tDCS of the lesioned side is in agreement with previous findings (Cuypers et al., 2013). Such effect can be linked to the increased excitability of the residual corticofugal projections from the lesioned hemisphere (Cuypers et al., 2013).

Additionally, for the first time, we found a non-significant effect of a-tDCS of the lesioned side when a table supported the paretic arm or a heavy SABD load was applied when reaching. For the table condition, there was no need to voluntarily recruit the shoulder abductor muscles while reaching. The residual contralateral cortical resources may thus be enough for performing the reaching task, which is in agreement with previous results showing that with table support, an individual with moderate to severe stroke can almost reach the full distance (Sukal et al., 2007; Ellis et al., 2009). In contrast, when a heavier SABD load was applied to the paretic arm, the residual cortical resources from the lesioned hemisphere may be insufficient. At this load, the effect of a-tDCS-induced increase in excitability of residual corticofugal projections from the lesioned side may be negligible when compared to the total amount of neural resources required. Therefore, recruitment was still mainly via the non-lesioned side. This may explain why an effect of a-tDCS was not found in our study when a heavy load (MVT_50) was applied.

### Effects of c-tDCS of Non-Lesioned Side on Reaching Distance with Various SABD Loadings

In our study, c-tDCS of the non-lesioned side significantly decreased the reaching distance when a heavy SABD load (MVT_50) was applied to the paretic arm. These results were in agreement with previous findings that for more severely impaired individuals, c-tDCS of the non-lesioned side failed to increase the excitability of the projections from the lesioned side (Bradnam et al., 2012). The significant effect of c-tDCS of the non-lesioned side was not shown when a table supported the paretic arm or a moderate load (MVT_25) was applied when reaching.

The exact reason for decreased reaching ability with heavy loading following c-tDCS of the non-lesioned hemisphere is not yet clear. One possible reason is that c-tDCS inhibits activity from the specific cortical area under the electrode, thus other cortical areas are recruited in order to perform the reaching task. Increased activity of other cortical regions may cause increased overlap between active cortical areas for different joins and thus increase synergy (Yao et al., 2009).

### Effects of a-tDCS of Non-Lesioned Side on Reaching Distance with Various SABD Loadings

Results obtained by applying a-tDCS to the non-lesioned side are not significantly different from those obtained by sham stimulation, regardless of shoulder loading. This may suggest that the cortical resources in the stimulated non-lesioned cortical area have already been fully utilized for the reaching task in individuals with moderate to severe stroke. An increase in activation of the non-lesioned hemisphere would, therefore, provide no additional functional benefit or detriment.

### Limitations

Our results are only based on results for 9 individuals with moderate to severe stroke. Further investigation with a larger number of participants will be required to validate these results. Furthermore, we only tested a single dosage of tDCS with fixed electrode size and position. Results of increased or reduced dosage of stimulation with different electrode size and position can be different (Rampersad et al., 2014). Lastly, different motor tasks may also change the effects of tDCS. For example, in this study, participants were required to perform the reaching task at a slow speed. Having participants performing ballistic reaching, which will increase the use of remaining neural resources, may change the results, and generate a more significant tDCS effect.

### Conclusion and Possible Clinical Impactions

In conclusion, results of this study, for the first time, show that the impact of tDCS on the expression of synergy is dependent on SABD loads in individuals with moderate to severe stroke. Optimal SABD loading should be combined with a-tDCS to the lesioned hemisphere to facilitate the usage of remaining corticospinal tracts from the lesioned hemisphere in individuals with moderate to severe stroke.

## Conflict of Interest Statement

The authors declare that the research was conducted in the absence of any commercial or financial relationships that could be construed as a potential conflict of interest.
